# Carbon Ion Beam Boost Irradiation in Malignant Tumors of the Nasal Vestibule and the Anterior Nasal Cavity as an Organ-Preserving Therapy

**DOI:** 10.3389/fonc.2022.814082

**Published:** 2022-02-15

**Authors:** Fabian Eberle, Rita Engenhart-Cabillic, Markus M. Schymalla, Christoph Dumke, Ulrike Schötz, Florentine S.B. Subtil, Kilian-Simon Baumann, Boris A. Stuck, Christine Langer, Alexandra D. Jensen, Henrik Hauswald, Stefan Lautenschläger

**Affiliations:** ^1^ Department of Radiation Oncology, Marburg University Hospital, Marburg, Germany; ^2^ Marburg Ion-Beam Therapy Center (MIT), Department of Radiation Oncology, Marburg University Hospital, Marburg, Germany; ^3^ Department of Otolaryngology/Head & Neck Surgery, Marburg University Hospital, Marburg, Germany; ^4^ Department of Otolaryngology/Head & Neck Surgery, Gießen University Hospital, Gießen, Germany; ^5^ Department of Radiation Oncology, Gießen University Hospital, Gießen, Germany; ^6^ Department of Radiation Oncology, Heidelberg University Hospital, Heidelberg, Germany

**Keywords:** malignancy of the nasal vestibule, malignancy of the nasal cavity, organ-preserving therapy, irradiation, carbon ion beam therapy, particle beam therapy, radiotherapy, nasal cancer

## Abstract

**Background:**

Surgery and radiotherapy are current therapeutic options for malignant tumors involving the nasal vestibule. Depending on the location, organ-preserving resection is not always possible, even for small tumors. Definitive radiotherapy is an alternative as an organ-preserving procedure. Carbon ion beam radiotherapy offers highly conformal dose distributions and more complex biological radiation effects eventually resulting in optimized normal tissue sparing and improved outcome. The aim of the current study was to analyze toxicity, local control (LC), and organ preserving survival (OPS) after irradiation of carcinoma of the nasal vestibule with raster-scanned carbon ion radiotherapy boost (CIRT-B) combined with volumetric intensity modulated arc therapy (VMAT) with photons.

**Methods:**

Between 12/2015 and 05/2021, 21 patients with malignant tumors involving the nasal vestibule were irradiated with CIRT-B combined with VMAT and retrospectively analyzed. Diagnosis was based on histologic findings. A total of 17 patients had squamous cell carcinoma (SCC) and 4 had other histologies. In this series, 10%, 67%, and 24% of patients had Wang stages 1, 2, and 3 tumors, respectively. Three patients had pathologic cervical nodes on MRI. The median CIRT-B dose was 24 Gy(RBE), while the median VMAT dose was 50 Gy. All patients with pathologic cervical nodes received simultaneously integrated boost with photons (SIB) up to a median dose of 62.5 Gy to the pathological lymph nodes. Eight patients received cisplatin chemotherapy. All patients received regular follow-up imaging after irradiation. Kaplan–Meier estimation was used for statistical assessment.

**Results:**

The median follow-up after irradiation was 18.9 months. There were no common toxicity criteria grade 5 or 4 adverse events. A total of 20 patients showed grade 3 adverse events mainly on skin and mucosa. All patients were alive at the end of follow-up. The median OPS after treatment was 56.5 months. The 6- and 24-month OPS were 100% and 83.3%, respectively. All local recurrences occurred within 12 months after radiotherapy. The median progression free survival (PFS) after treatment was 52.4 months. The 6-, 12-, and 24-month PFS rates were 95%, 83.6%, and 74.3%, respectively.

**Conclusion:**

CIRT-B combined with VMAT in malignant tumors of the nasal vestibule is safe and feasible, results in high local control rates, and thus is a good option as organ-preserving therapy. No radiation-associated grade 4 or 5 acute or late AE was documented.

## Introduction

Malignant tumors of the nasal vestibule and the anterior nasal cavity are rare and account for less than 1% of all head and neck tumors (1, 2). Primary tumors of the nasal vestibule had an estimated standardized incidence of 0.4 per 100,000 inhabitants (3). There are three main staging systems: the American Joint Committee on Cancer (AJCC) (4), the Union for International Cancer Control (UICC) (5), and the Wang system (6) (**Table 1**). The Wang classification is a staging system based primarily on clinical tumor characteristics. It is considered the most appropriate classification system for malignancy of the nasal vestibule (7–9). Standard of care includes surgery, with or without adjuvant radiotherapy in certain postoperative risk constellations or definitive radiotherapy. Although surgery can yield high control rates, organ preservation may not always be possible, even for small tumors (10–12). Definitive radiotherapy for malignant tumors of the nasal vestibule and the anterior nasal cavity involving the nasal vestibule may be preferable as an organ-preserving procedure (13). Different irradiation techniques such as brachytherapy (9, 14, 15) or external beam radiotherapy (EBRT) (8, 16) or a combination of both (17) are available. Especially in early stages, any of these treatment options leads to high local control rates and can yield good cosmetic and functional results. For larger lesions, control rates decrease after definitive EBRT with photons (8). Carbon ions have different radiobiological effects eventually being able to overcome radioresistance (18, 19). For example, carbon ions could eradicate hypoxic and stem cell-like tumor cells and create an antiangiogenic and less immunosuppressive state (20, 21). Furthermore, due to the specific energy deposition resulting in the Bragg-Peak, carbon ions offer improved normal tissue sparing. Therefore, carbon ion beam radiotherapy might be more effective in eliminating tumor cells while showing less adverse events compared to photon beam radiotherapy. Currently, there are no clinical data on radiotherapy with carbon ion boost (CIRT-B) combined with volumetric intensity modulated arc therapy (VMAT) for tumors of the nasal vestibule and the anterior nasal cavity. For other tumors in the head and neck region, excellent results have been achieved with the use of carbon ions (22–26). The aim of the current study was to analyze toxicity, local control, and organ-preserving survival after irradiation of malignant tumors of the nasal vestibule and the anterior nasal cavity with raster-scanned CIRT-B combined with VMAT with photons as organ-preserving therapy at the Marburg Ion-Beam Therapy Center/Marburg University Hospital.

**Table 1:** Classification systems for malignancies of the nasal cavity/paranasal sinuses and the nasal vestibule.

	American Joint Committee on Cancer (AJCC)	Union International Centre le Cancer (UICC 2002)	Wang-classification for malignancy of the nasal vestibule
**T1**	Tumor restricted to any 1 subsite, with or without bony invasion	Limited to 1 subsite	Limited to the nasal vestibule, relative superficial, involving 1 or more sites within
**T2**	Tumor invading 2 subsites in a single region or extending to involve an adjacent region within the nasoethmoidal complex, with or without bony invasion	Involves 2 subsites or adjacent nasoethmoidal site	Extended from the nasal vestibule to adjacent structures, such as the upper nasal septum, upper lip, philtrum, skin of the nose, and/or nasolabial fold, but not fixed to the underlying bone
**T3**	Tumor extends to invade the medial wall or floor of the orbit, maxillary sinus, palate, or cribriform plate	Invasion of medial wall/floor orbit, maxillary sinus, palate,cribriform plate	Massive with extension to the hard palate, buccogingival sulcus, large portion of the upper lip, upper nasal septum, turbinate, and/or paranasal sinuses, fixed with deep muscle or bone involvement
**T4a**	Tumor invades any of the following: anterior orbital contents, skin of the nose or cheek, minimal extension to the anterior cranial fossa, pterygoid plates, sphenoid or frontal sinuses	Involvement of anterior orbit, skin of nose/cheek, anterior cranial fossa, pterygoid plates,sphenoid/frontal sinuses	undefined
**T4b**	Tumor invades any of the following: orbital apex, dura, brain, middle cranial fossa, cranial nerves other than maxillary division of trigeminal nerve (V2), nasopharynx, or clivus	Involvement of orbital apex, dura, brain, middle cranial fossa, cranial nerves other than V2, nasopharynx, clivus	undefined

## Materials and Methods

### Patients’ Characteristics

Between November 2015 and May 2021, 21 patients from Marburg and Gießen University Hospital mainly with SCC of the nasal vestibule and the anterior nasal cavity were irradiated at the Marburg Ion-Beam Therapy Center with CIRT-B combined with VMAT carried out at the Department of Radiation Oncology of the Marburg University Hospital. Diagnosis was primarily based on histologic findings and on magnetic resonance imaging (MRI). Further patients’ and treatment characteristics are found in **Tables 2**, **3**.

**Table 2:** Patients’ characteristics.

Parameter	N	%
**Gender**		
Male	12	57
Female	9	43
**Age, years**		
Median	57	
Range	44-89	
**ECOG Score at RT**		
0	17	81
1	4	19
Smoking history		
Smoker	7	34
Non-Smoker	14	66
**Histology**		
SCC	17	81
Others (AC, AS, MYC, MEC)	4	9
**Grading**		
G1	3	14
G2	11	52
G3	7	33
**HPV status (p16)**		
negative	6	29
positive	2	10
n.a.	13	61
**Tumor site**		
vestibule	2	10
vestibule and anterior nasal cavity	19	90
**Largest diameter, mm**		
Median	22.5	
Range	14-43	
**PT Stage**		
Wang		
1	2	9
2	14	67
3	5	24
AJCC		
1	2	10
2	15	71
3	-	-
4a	4	19
4b	-	-
UICC		
1	2	10
2	15	71
3	-	-
4a	4	19
4b	-	-
**Nodal stage**		
N0	18	86
N1	-	-
N2a	-	-
N2b	1	5
N2c	2	9
**Skin invasion**		
Yes	4	19
No	17	81
**Bone invasion**		
Yes	3	14
No	18	86

SCC, squamous cell carcinoma; AC, adenocarcinoma; AS, angiosarcoma; MYC, myoepithelial carcinoma; MEC, mucoepidermoid carcinoma; n.a., not available; RT, radiotherapy; PT, primary tumor.

**Table 3:** Treatment characteristics.

Parameter	N	%
**RT setting**		
primary	19	90
salvage	2	10
**Resection performed (before RT)**		
Yes	2	10
No	19	90
**Intervall between resection and RT, months**		
Median	24.5	
Range	19-30	
**Dose C_12_ Boost, Gy(RBE)**		
Total dose (median)	24	
Range	18-24	
Single dose	3	
**GTV Boost, ccm**		
Median	4.1	
Range	1.2-26.4	
**CTV Boost, ccm**		
Median	171.3	
Range	7.3-	
**PTV Boost, ccm**		
Median	28.8	
Range	1	
**Dose Photons, Gy**		
Total dose (median)	50	
Range	50-56	
Single dose	2	
**CTV Photons, ccm**		
Median	171.3	
Range	7.3-436.6	
**ENI performed**		
Yes	14	67
No	7	33
**Platinbased chemotherapy administered**		
Yes	8	38
No	13	62
**Duration of RT, days**		
Median	48.5	
Range	38-52	

SCC, squamous cell carcinoma; AC, adenocarcinoma; AS, angiosarcoma; MYC, myoepithelial carcinoma; MEC, mucoepidermoid carcinoma; n.a., not available; RT, radiotherapy; PT, primary tumor; Gy(RBE), Gray (relative biological effectiveness); Gy, Gray; GTV, gross tumor volume; CTV, clinical target volume; PTV, planning target volume; Ccm, cubic centimeter; ENI, elective node irradiation; C_12_, carbon ions.

### Initial Treatment

Two patients underwent organ-preserving surgery at initial diagnosis. Due to the early stages and missing evidence of tumor after surgery, no adjuvant treatment was performed. Macroscopic recurrence occurred during regular oncologic follow-up, and definitive salvage RT was performed. For these two patients, the time interval between resection and diagnosis of recurrent disease was 19 and 30 months, respectively. No patient received prior chemotherapy or radiotherapy.

### Immobilization and Target Volume Definition

For patient immobilization, a thermoplastic head-shoulder-mask was used. Computed tomography (CT, 3‐mm slices) was used for treatment planning. For precise contouring, a T1-weighted contrast-enhanced MRI was three‐dimensionally registered to the planning CT. The gross tumor volume (GTV-B) was defined as the contrast-enhancing primary tumor on a T1 contrast-enhanced MRI. If there was nodal involvement, a second GTV (GTV-SIB) was delineated. Separate clinical target volumes (CTVs) were delineated. The clinical target volume for the CIRT boost (CTV-B) was defined as a 5-mm expansion to the GTV-B respecting anatomical borders. CTV-photons were the extended target volume and included CTV-B, typical pathways of spread, and in advanced stages and in patients with nodal involvement elective lymph node levels (facial, Ib, II, III). The clinical target volume for SIB (CTV-SIB) was defined as a 5- to 7-mm expansion of the GTV-SIB. The planning target volume (PTV) was defined as the CTV plus a 3-mm margin.

### Treatment Planning

Treatment planning for raster-scanned CIRT-B was performed with the Siemens Syngo.via PT planning software. Biological dose optimization was performed based on the local effect model (LEM) 1. VMAT plans were calculated with the Varian ECLIPSE V 15.6 planning software.

### Treatment

CIRT-B was performed at the Marburg Ion-Beam Therapy Center with carbon ion (^12^C) beams *via* the active raster scanning method with 2 to 4 noncoplanar treatment beams under daily image guidance with orthogonal X-rays and weekly CT-based recalculations. Photon treatment was carried out in Rapid Arc IMRT technique at the Department of Radiation Oncology of the Marburg University Hospital. A Varian True Beam linear accelerator with a motoric multileaf collimator of 0.5-cm leaf width under daily image guidance with CBCT in the treatment position was used. The prescribed dose was normalized to the median dose of the target volume. Furthermore, the PTV was encompassed within the 95–107% isodose level of the prescribed dose. Patients received a CIRT-B with 18–24 Gy(RBE) to PTV-B in 6–8 fractions followed by 50–56 Gy photon VMAT to PTV-photons in 2 Gy per fraction. Patients with nodal involvement received simultaneously integrated photon boost (SIB) up to 62.1–64.4 Gy with 2.3 Gy per fraction to PTV-SIB. Five fractions per week were administered.

In advanced stages and in patients with nodal involvement on MRI elective nodal irradiation (ENI) with simultaneously integrated boost (SIB) was performed with photons and cisplatin chemotherapy was administered simultaneously to photon treatment (40 mg/m^2^ weekly). Further treatment characteristics are found in **Table 3**.

### Evaluation

Prospectively collected datasets and medical reports of all patients who received irradiation treatment with CIRT-B followed by VMAT between 2015 and 2021 for malignancies of the anterior nasal cavity with involvement of the nasal vestibule were evaluated. Treatment and follow-up was performed according to a fixed scheme at our center. The first clinical follow-up examination was 6 weeks after finishing radiotherapy; the first follow-up examination including MRI of the head and neck was 3 months after finishing radiotherapy and every 3 months thereafter. Adverse events (AEs) were classified according to the common toxicity criteria for adverse events version 5 (CTCAE V.5).

### Statistical Design and Classifications

Toxicity, organ-preserving survival (OPS), local control (LC), and progression-free survival (PFS) were evaluated. Time estimates refer to the date of treatment planning CT. LC was defined as the absence of local tumor progression including all cases of stable disease (less than 50% tumor mass reduction), partial remission (tumor mass reduction of at least 50%), and complete remission (requiring no detectable disease). Survival analyses were carried out with I.B.M. SPSS 21 using Kaplan–Meier estimation and log rank test.

### Ethics

The local ethics committee approved the study (Marburg, Germany, study number EK_MR_31_03_21). All patients gave informed consent. This study was conducted in accordance with the Declaration of Helsinki.

### Data Sharing Statement

Due to the legal aspects of the patients’ informed consent, sharing of data is not possible.

## Results

### Adverse Events

**Figure 1:**
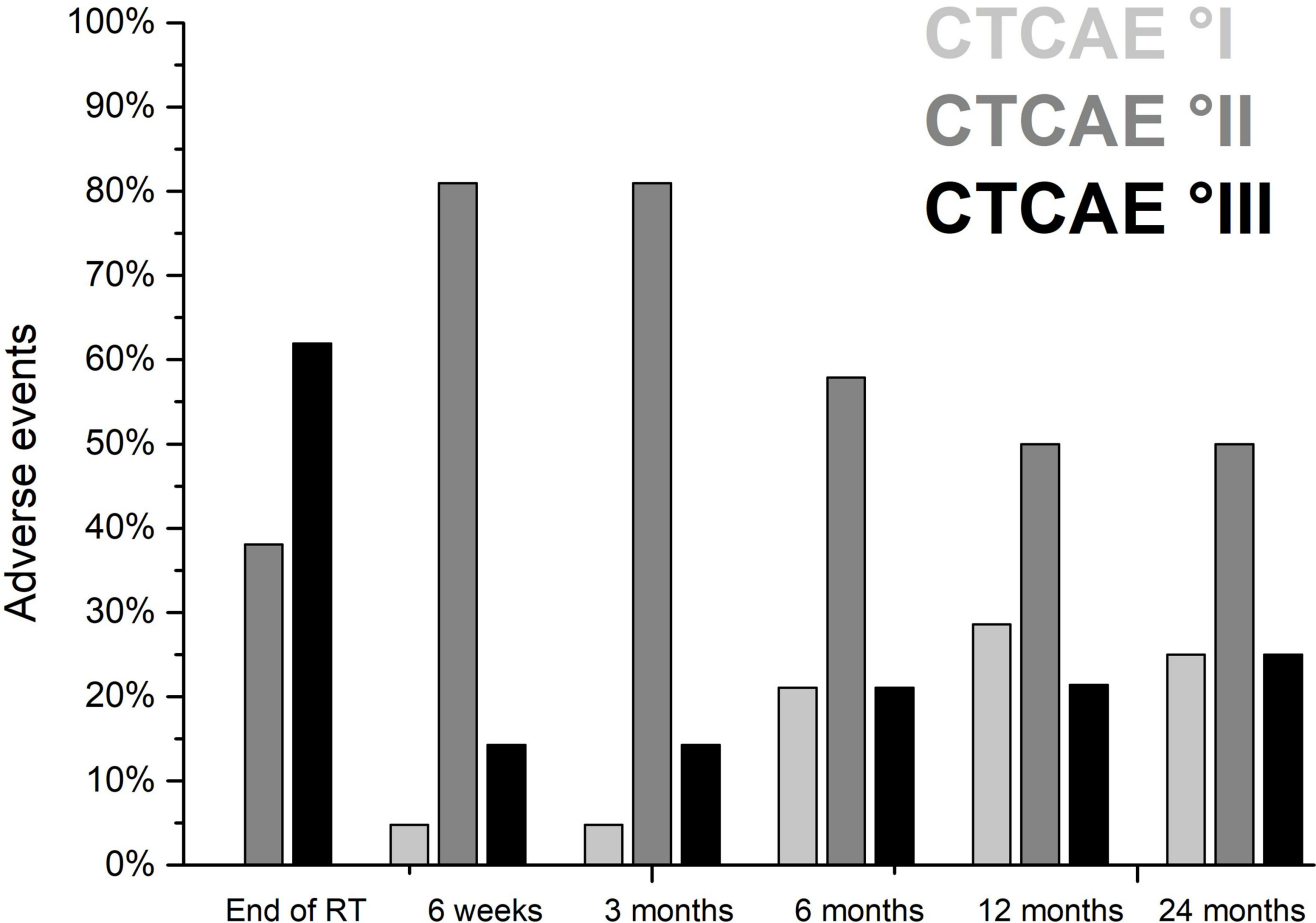
Acute and late adverse events after definitive radiotherapy of 21 patients with malignant tumors involving the nasal vestibule irradiated with CIRT-B combined with VMAT according to common toxicity criteria for adverse events (CTCAE V 5.0).

According to CTCAE V 5.0, none of the patients developed CTC grade 5 or 4 AE. At the end of treatment, 61.9% of the patients developed grade 3 and 38.1% of the patients developed grade 2 acute AE mostly on skin, mucosa, and on swallowing. Rapid recovery from skin and mucosal toxicity was seen in the majority of the patients. Six weeks after completion of treatment, 14.3% of the patients showed grade 3 acute AE. CTC grades 1 and 2 acute AE at the end of treatment and 6 weeks after treatment were seen in 38.1% and 81.0% of the patients, respectively (**Figure 1** and **Table 4**).

**Table 4:** Treatment-related acute and late adverse events according to common toxicity criteria for adverse events (CTCAE V 5.0).

	End of RT			6 weeks			3 months			6 months			12 months			24 months		
CTCAE grade	I	II	III	I	II	III	I	II	III	I	II	III	I	II	III	I	II	III
Number of patients at FU	n=21			n=21			n=21			n=19			n=14			n=8		
**Dermatitis [%]**	0.0	47.6	52.4	85.7	14.3	0.0	28.6	4.8	0.0	26.3	0.0	0.0	7.1	0.0	0.0	0.0	0.0	0.0
**Mucositis [%]**	19.0	23.8	52.4	38.1	28.6	0.0	19.0	4.8	0.0	10.5	0.0	0.0	0.0	0.0	0.0	0.0	0.0	0.0
**Dysphagia [%]**	19.0	33.3	33.3	28.6	33.3	9.5	42.9	14.3	4.8	15.8	5.3	0.0	14.3	0.0	0.0	0.0	0.0	0.0
**Dysgeusia [%]**	19.0	66.7	undefined	33.3	52.4	undefined	47.6	23.8	undefined	52.6	10.5	undefined	50.0	14.3	undefined	37.5	0.0	undefined
**Dry mouth [%]**	9.5	52.4	28.6	14.3	66.7	9.5	19.0	66.7	4.8	36.8	47.4	0.0	64.3	21.4	0.0	50.0	12.5	0.0
**Dry eye [%]**	52.4	0.0	0.0	38.1	0.0	0.0	19.0	0.0	0.0	10.5	0.0	0.0	14.3	0.0	0.0	12.5	0.0	0.0
**Optic nerve disorder [%]**	0.0	0.0	0.0	0.0	0.0	0.0	0.0	0.0	0.0	0.0	0.0	0.0	0.0	0.0	0.0	0.0	0.0	0.0
**Hearing impaired [%]**	4.8	4.8	0.0	4.8	4.8	0.0	4.8	9.5	0.0	5.3	5.3	5.3	7.1	7.1	7.1	0.0	0.0	12.5
**Epistaxis [%]**	42.9	0.0	0.0	28.6	0.0	0.0	19.0	0.0	0.0	15.8	0.0	0.0	14.3	0.0	0.0	12.5	0.0	0.0
**Sinus disorders [%]**	23.8	52.4	23.8	23.8	71.4	4.8	47.6	33.3	9.5	57.9	15.8	15.8	64.3	14.3	14.3	75.0	0.0	12.5
**Soft tissue fibrosis [%]**	9.5	0.0	0.0	19.0	0.0	0.0	28.6	23.8	0.0	21.1	47.4	0.0	14.3	50.0	0.0	12.5	50.0	0.0

The most frequent acute AE CTC grade 3 at the end of treatment were dermatitis, dry mouth with inability to adequately aliment orally, mucositis with severe pain affecting oral intake, and dysphagia with severely altered eating/swallowing in 52.4%, 28.6%, 52.4%, and 33.3% of the patients, respectively. Inpatient treatment of patients with mucosal AE and impaired swallowing during radiotherapy was required in 8 patients (38.1%). Tube feeding was indicated in 7 patients (33.3%). In the CTCAE classification (V 5.0), there is no separate category for therapy-related complaints in the area of the nasal vestibule. Complaints in this region are thus best represented within the CTCAE term “sinus disorders.” Sinus disorders CTCAE grade 2 with impairment of airflow or CTCAE grade 3 with significant nasal obstruction occurred in 52.4% and 23.8% of the patients, respectively. One case of cisplatin-related hearing loss without indication for intervention or hearing aid fitting occurred. At the end of treatment, CTCAE grade 1 dry eyes with mild symptoms relieved by lubricants were common in 52.4% of the patients. Grade 1 epistaxis without indication for intervention was seen in 42.9% of the patients.

Rapid and extensive recovery from skin and mucosal toxicity, xerostomia, dysphagia, and sinus disorders were observed in a majority of patients. Six weeks after completion of treatment, residual dry mouth or dysphagia and residual sinus disorders CTCAE grade 3 were present in 9.5% and 4.8% of the patients, respectively.

Late AE CTC grade 3 occurred in 14.2% (after 3 months), 21.1% (after 6 months), 21.4% (after 12 months), and 25.0% (after 24 months) of patients mostly consisting of nasal obstruction or cisplatin related hearing impairment that required medical intervention. In our series, 80.9%, 73.7%, 78.6%, and 75% of the patients had grade 1 or 2 sinus disorders with mucosal crusting or symptomatic stenosis at the level of the nasal vestibule interfering with airflow 3, 6, 12 and 24 months after radiotherapy, respectively. Additional three patients developed CTCAE grade 3 stenosis with significant nasal obstruction and limited airflow at the level of the nasal vestibule within the first 3–6 months after completion of radiotherapy, which required intervention. These limitations were most likely due to the formation of synechiae at the level of the nasal vestibule caused by therapy-related mucosal ulceration. Surgical intervention with removal of adhesions restored good airflow and respiratory function. One patient receiving radiochemotherapy developed cisplatin-related CTCAE grade 3 hearing loss 6 months after treatment, and a bilateral hearing aid was needed. CTC grades 1 and 2 AE 3, 6, 12, and 24 months after treatment were seen in 80.9%, 57.9%, 50.0%, and 50.0% of the patients, respectively (**Figure 1** and **Table 4**). Fibrotic changes CTCAE grade 2 of the soft tissue occurred at the earliest 3 months after the end of therapy. After 3, 6, 12, and 24 months, 23.8%, 47.4%, 50.0%, and 50.0% of the patients showed therapy-related fibrotic processes CTCAE grade 2 of the nasal soft tissue, respectively. No patient developed cartilage necrosis during follow-up. One patient with nose piercing developed a small soft tissue necrosis of the ala nasi, which required local wound care. No surgical intervention was required in this case. Six months after the end of treatment, there was no CTCAE grade 3 dysphagia or dry mouth. However, moderate dry mouth CTCAE grade 2 persisted after 6, 12, and 24 months in 47.4%, 21.4%, and 12.5% of the patients, respectively. Altered taste/unpleasant taste was present after 6 and 12 months in 2 patients. Further parameters regarding acute and late AE are found in **Table 4**.

### Local Control and Survival

**Figure 2:**
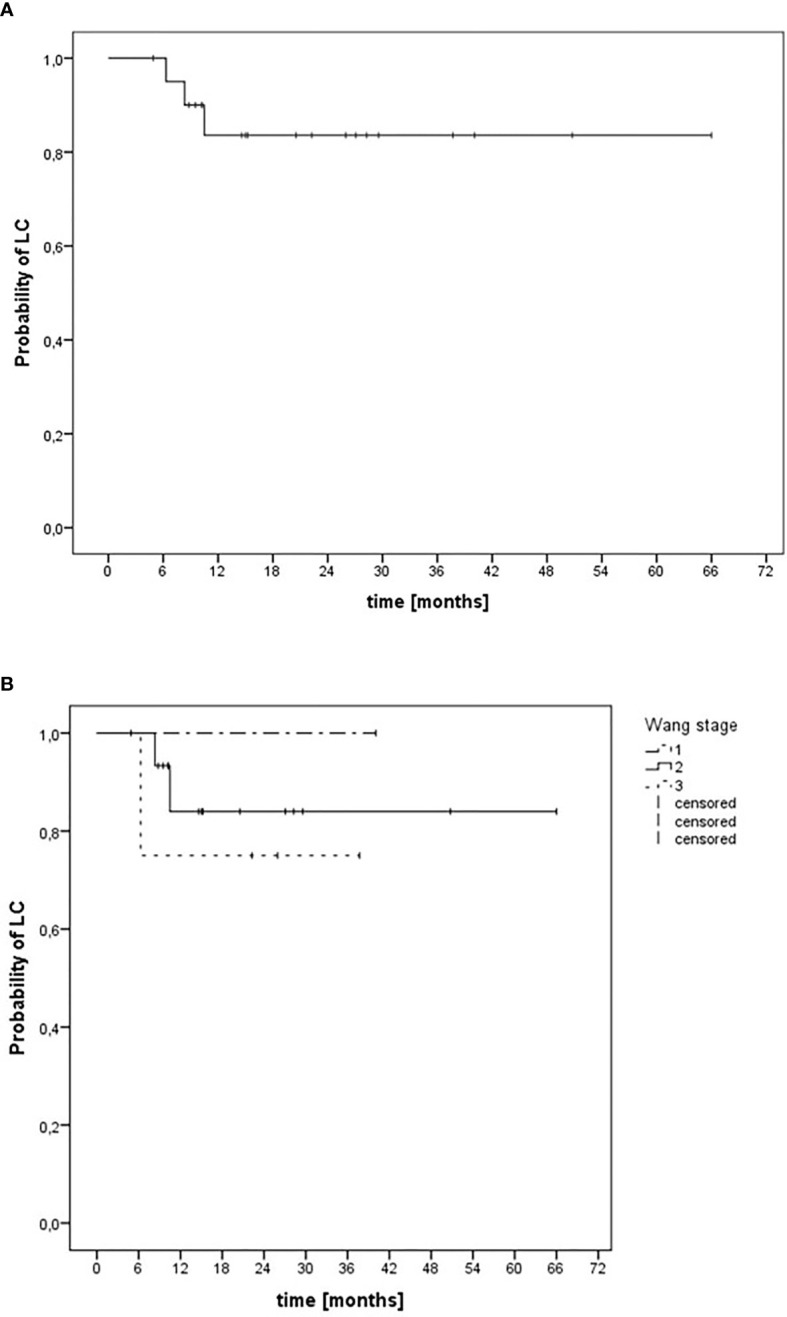
Kaplan–Meier estimation of local control after definitive radiotherapy of 21 patients with malignant tumors involving the nasal vestibule irradiated with CIRT-B combined with VMAT. **(A)** LC independent of tumor stage. **(B)** LC depending on Wang stage.

The median follow-up after treatment was 18.9 months (range, 3–64 months). All patients were alive at the end of follow-up. The estimated median LC after diagnosis was 56 months (range, 46–66 months). The actuarial 24-month LC rates after diagnosis for all patients (**Figure 2A**) and patients with Wang stage 3 tumors (**Figure 2B**) were 84% and 75%, respectively. Eighty percent of the patients showed complete clinical response without evidence of tumor on MRI 3 months after radiotherapy. There were three patients with local tumor progression after treatment. In all patients, this occurred within the first 12 months after therapy. Out-of-field (CIRT-B) progression of a single submandibular node was seen in one patient. In field (CIRT-B) progression was seen in two patients. Median time to progression at the initial tumor site was 6 months (range, 4–8 months). Two patients with local tumor progression underwent non organ-preserving salvage surgery; in one patient, organ-preserving salvage resection was feasible. Median time to salvage surgery after finishing initial treatment was 6 months (range, 5–10 months). None of the patients who had undergone salvage surgery developed tumor recurrence during further follow-up.

**Figure 3:**
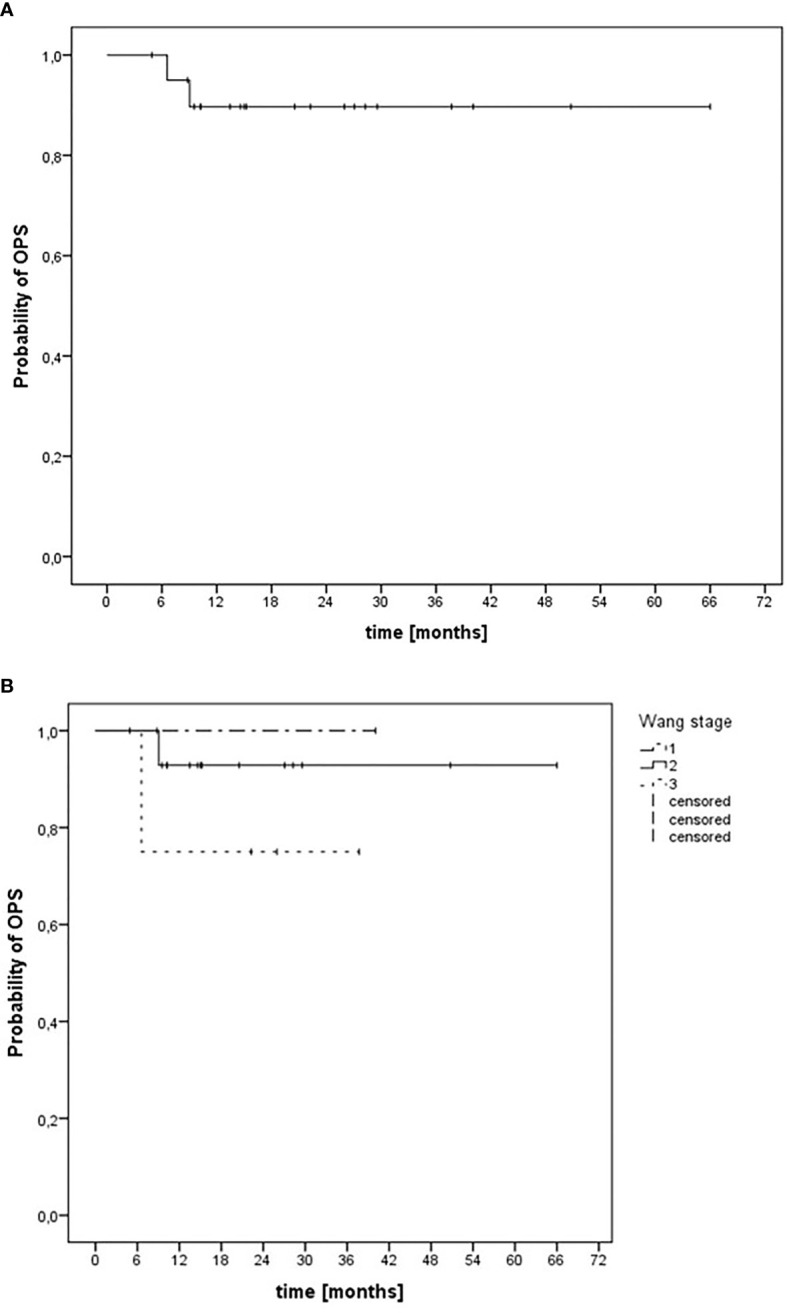
Kaplan–Meier estimation of organ-preserving survival (OPS) after CIRT-B combined with VMAT. **(A)** OPS independent of tumor stage. **(B)** OPS depending on Wang stage.

For malignant tumors of the nasal vestibule and the anterior nasal cavity, the median organ-preserving survival (OPS) after diagnosis was 60 months (range, 52–68 months). The corresponding 6- and 12-month OPS rates after diagnosis for all patients were 100% and 90%, respectively (**Figure 3A**). For patients with Wang stage 3 tumors, the 12-month OPS rate was 75% (**Figure 3B**).

**Figure 4:**
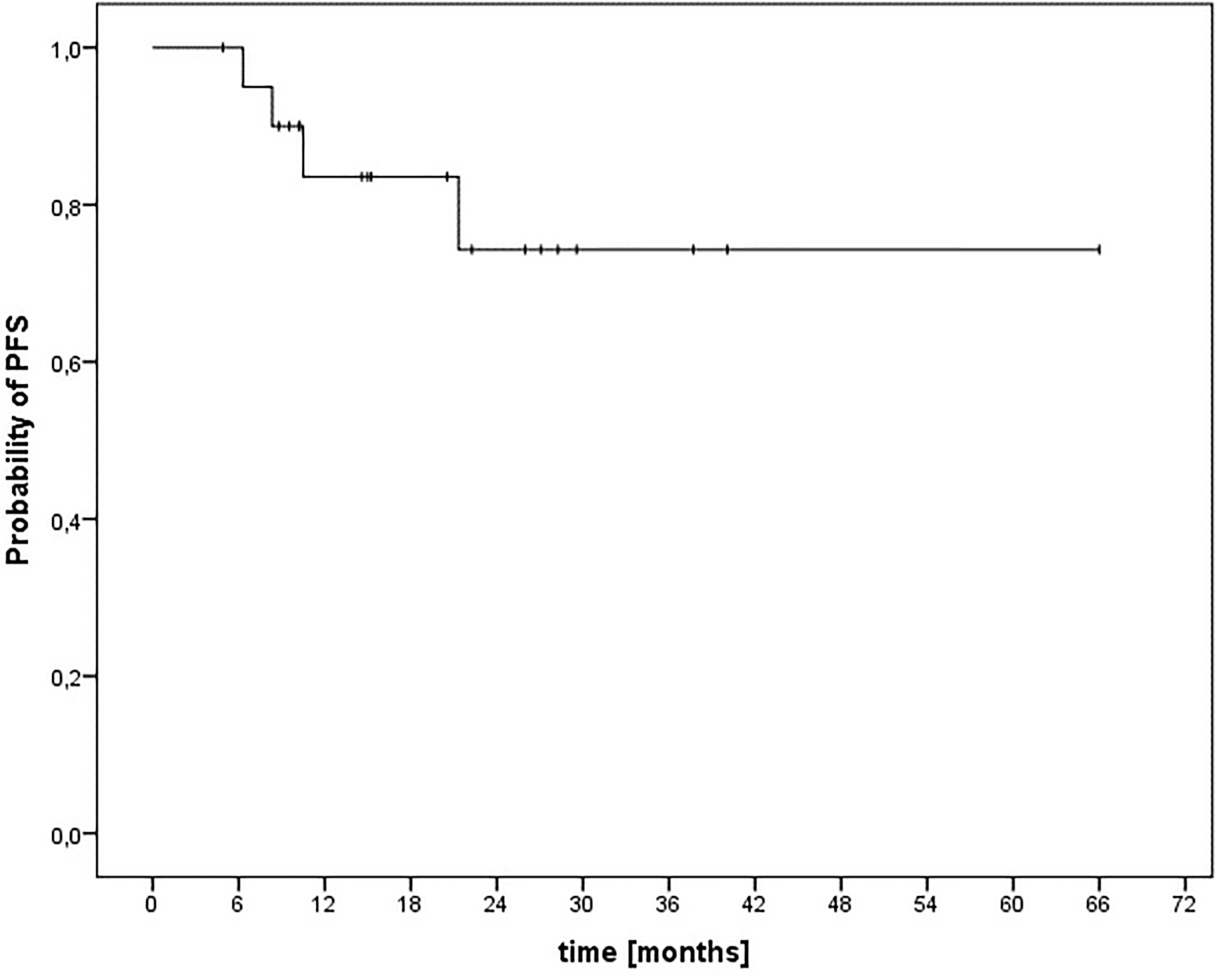
Kaplan–Meier estimation of progression-free survival after CIRT-B combined with VMAT.

The median PFS after diagnosis was 52 months (range, 40–64 months). The corresponding 12- and 24-month PFS rates after diagnosis were 84% and 74%, respectively (**Figure 4**). There was one patient with locoregional relapse of a single submandibular node without evidence of tumor at the primary site. Initial treatment was performed as a local radiotherapy without ENI and without chemotherapy due to tumor stage. Nodal relapse occurred 19 months after end of radiotherapy. Salvage surgery followed by adjuvant elective irradiation of the lymphatic drain was performed. This patient remained free of tumor until the end of follow-up.

### Prognostic Factors for LC

In univariate analysis on factors impacting on LC histology (SCC versus others, p=0.44), Wang stage (all stages, p=0.77), AJCC stage (all stages, p=0.71), UICC stage (all stages, p=0.72), size of GTV (>3.9 ccm versus <3.9 ccm, p=0.46), size of CTV (>8.2 ccm versus <8.2 ccm, p=0.83), size of PTV (>31.8 ccm versus <31.8 ccm, p=0.24), maximal tumor diameter (>20 mm versus <20 mm, p=0.28), presence of bone infiltration (yes versus no, p=0.31), presence of skin infiltration (yes versus no, p=0.51), upper lip involvement (yes versus no, p=0.66), upper septum involvement (yes versus no, p=0.86), delivery of chemotherapy (delivery versus no delivery, p=0.53), and previously tumor resection (yes versus no, p=0.15) did not demonstrate statistically significant effects. Further parameters are demonstrated in **Table 5**. Multivariable analysis on LC was not performed due to missing prognostic factors in univariate analysis and the limited number of patients.

**Table 5:** Univariate analyses on local control (Log Rank Test).

Parameter	p-value
Smoker	0.82
Gender	0.83
Age (>56.4 years)	0.59
Histology	
Histology (SCC versus others)	0.44
Grading	0.33
HPV status	0.51
Stage	
Wang (all stages)	0.77
Wang (3 versus 1)	0.62
Wang (3 versus other stages)	0.53
AJCC (all stages)	0.71
AJCC (4 versus 1)	0.48
AJCC (4 versus other stages)	0.52
UICC (all stages)	0.72
UICC (4 versus 1)	0.48
UICC (4 versus other stages)	0.53
Tumor size	
GTV size (>3.9ccm)	0.46
CTV size (>8.2ccm)	0.83
PTV size (>31.8ccm)	0.24
Tumor diameter (>20mm)	0.28
Clinical parameters	
Presence of skin infiltration	0.51
Presence of bone infiltration	0.31
Upper lip involvement	0.66
Upper septum involvement	0.86
Delivery of chemotherapy	0.53
Previously tumor resection	0.15

*p < 0.05.

GTV, gross tumor volume; CTV, clinical target volume; PTV, planning target volume; SCC, squamous cell carcinoma.

### Prognostic Factors for Survival

Histology (SCC versus others, p=0.50), tumor stage (Wang stage, p=0.50; AJCC stage, p=0.48); UICC stage, p=0.49), and presence of bone invasion (yes versus no, p=0.12) did not demonstrate statistically significant effects on OPS. Furthermore, target volume and tumor size had no statistically significant effect on OPS (GTV>3.9 ccm, p=0.54; CTV>8.2 ccm, p=0.83; PTV>31.8 ccm, p=0.62; maximal tumor diameter>20 mm, p=0.76). Additional parameters are found in **Table 6**. Multivariable analysis on survival was not performed due to missing prognostic factors in univariate analysis.

**Table 6:** Univariate analyses on organ-preserving survival (OPS) (Log Rank Test).

Parameter	p-value
Smoker	0.56
Gender	0.25
Age (>56.4 years)	0.16
Histology	
Histology (SCC versus others)	0.50
Grading	0.12
HPV status	0.56
Stage	
*Wang (all stages)*	*0.50*
Wang (3 versus 1)	0.61
Wang (3 versus other stages)	0.25
*AJCC (all stages)*	*0.48*
AJCC (4 versus 1)	0.48
AJCC (4 versus other stages)	0.24
*UICC (all stages)*	*0.49*
UICC (4 versus 1)	0.48
UICC (4 versus other stages)	0.25
Tumor size	
GTV size (>3.9ccm)	0.54
CTV size (>8.2ccm)	0.83
PTV size (>31.8ccm)	0.62
Tumor diameter (>20mm)	0.76
Clinical parameters	
Presence of skin infiltration	0.24
Presence of bone infiltration	0.12
Upper lip involvement	0.74
Upper septum involvement	0.58
Delivery of chemotherapy	1.00
Previously tumor resection	0.07

*p < 0.05.

GTV, gross tumor volume; CTV, clinical target volume; PTV, planning target volume; SCC, squamous cell carcinoma.

## Discussion

We have analyzed all patients with malignant tumors of the vestibule or the anterior nasal cavity with involvement of the nasal vestibule consecutively treated with CIRT-B combined with VMAT at Marburg Ion-Beam Therapy Center and Department of Radiation Oncology of the Marburg University Hospital between December 2015 and May 2021.

It was our aim to retrospectively assess the treatment results in our patients and help finding ways to improve the outcome in this rare and challenging disease. To our knowledge, this is the first report on clinical outcomes after irradiation with CIRT-B followed by photon EBRT in malignant tumors of the vestibule or the anterior nasal cavity with involvement of the nasal vestibule.

Langendijk et al. evaluated the results of primary RT for SCC of the nasal vestibule. A total of 56 patients with Stage T1 and T2 tumors (Wang classification) were treated. The 2-year LC-rate was 88% after EBRT. The 2-year locoregional control (LRC) was 87% (16). As with our patients, none of the patients developed distant metastases. Out of 10 patients with local recurrence at the primary tumor site, 8 were successfully salvaged by surgery. The ultimate local control rate after 5 years was 95%. In our collective ultimate LC and LRC following surgical salvage treatment (local (n = 3), locoregional (n = 1)) was 100% after a median follow-up for these patients of 18.2 months. In a retrospective analysis of 174 patients receiving surgery, radiotherapy, or both treatment modalities conducted by Agger et al., LC and disease-specific survival for all patients after 5 years were 80% and 74%, respectively (8). In a stratified analysis of T1 tumors (Wang), the authors found a higher 5-year LRC for surgery compared to the hypofractionated high-dose radiotherapy group (EQD2 67.5) (94% versus 87%). The subgroup of patients who were treated with RT doses below 66 Gy performed worse with LRC rates of 60% after 5 years. This suggests that for RT alone, a sufficiently high dose is crucial for the outcome of therapy. Vanneste et al. reported on 81 patients who were treated with EBRT (TD 59.4 Gy, SD 2.7) or interstitial brachytherapy (60 Gy) for primary, localized, SCC of the nasal vestibule. LC at 5 years over all stages was 85%; T1 tumors performed better with LC of 97% (17). Interventional radiotherapeutic (IR) procedures are mainly used for small tumors (Wang stages 1 and 2). Good clinical results with LC rates of 80–90% after 5 years can be achieved when IR for Wang stages 1 and 2 tumors is used. For all patients with local recurrence, salvage resection was possible and performed (9, 14, 27). Primary CIRT-B and EBRT treatment resulted in LC and PFS rates of 84% and 74% after 24 months, respectively, in our cohort. These results are in the range of those reported by others on primary RT in malignancy of the nasal vestibule. However, direct comparability with other published data is difficult due to the use of different treatment concepts, radiation techniques, staging systems, small and inhomogeneous patient collectives, and various endpoints.

There are no data on the treatment of malignant tumors of the nasal vestibule with carbon ions, but clinical data exist on the treatment of head and neck and paranasal sinus tumors with this irradiation technique. Studies showed that carbon ion radiotherapy can yield favorable outcomes for patients with certain head and neck tumors, e.g., adenoid cystic carcinoma, recurrent head and neck cancer, or mucosal melanoma (22–26). In a retrospective analysis of 95 patients with locally advanced adenoid cystic carcinoma of the head and neck, definitive raster-scanned C12 therapy was compared with modern photon techniques. LC, PFS, and OS at 5 years were significantly higher in the C12 group (59.6%, 48.4%, and 76.5%, respectively) compared with the photon group (39.9%, 27%, and 58.7%, respectively) (25). In a retrospective study, 229 patients with recurrent head and neck cancer were treated with carbon ion radiotherapy (CIRT). CIRT seems to be an effective treatment with acceptable toxicity resulting in good LC rates. Median local PFS and OS after radiotherapy with carbon ions were 24.2 and 26.1 months, respectively (28). In a retrospective analysis performed by Mohr et al., CIRT was used for the treatment of mucosal melanoma of the paranasal sinuses. LC at 3 years was 58.3% at mild toxicity. OS was poor due to the occurrence of distant metastases (29).

Surgical resection is often performed for malignancies of the nasal vestibule and is considered a reliable local treatment option especially for advanced stages or as salvage treatment (30). Resection, even if cosmetically compromising, can achieve high local control rates between 82% and 94% after 3 years similar to RT in appropriately selected patients (3, 8, 10, 31, 32). Depending on tumor location and extension, organ-preserving resection is not always possible, and in advanced stages, resection should be combined with postoperative RT (10, 15, 31–33).

In the report by Kummer et al., all 47 patients experienced acute RT AE, mainly dermatitis (28% °III), mucositis (30% °III), and crust. Acute mucosal and skin AE grade 3 occurred in 30% and 28% of the patients, respectively. Late radiation AE was reported only in a few patients. A perforated nasal septum due to cartilage necrosis occurred in three patients (6%); severe stenosis of the nasal airway was reported for two cases (4%) (34). In the cohort by Vanneste et al., all patients experienced acute dermatitis of the nasal skin and mucositis of the nasal cavity. Grade III mucositis of the oral cavity was seen in 10% of the patients. About 72% of the patients survived long-term without AE. Three patients (3%) experienced a perforated septum; in 2 cases, the nasal septum showed tumor infiltration. Two patients (2.5%) experienced severe stenosis of the nasal airway (17). The patients in the study of Wallace et al. mainly reported moderate soft tissue AE (21%) that resolved without intervention. Severe complications occurred in 4.2% of the patients treated with RT (35). In addition, Langendijk et al. reported that the most common late AE were rhinorrhea (45%), nasal dryness (39%), and adhesions (4%) (16). There was no CTC grade 5 or 4 AE, but 20 patients showed grade 3 adverse events mainly on the skin and mucosa. However, even though there was no grade 4 or 5 AE in our cohort, 20 patients developed CTC grade 3 acute AE, requiring medical intervention. The high rate of AE, especially at the skin and mucosa, is consistent with the data for RT of malignancies of the nasal vestibule mentioned above. In our cohort, relevant stenoses CTCAE grade 3 at the level of the nasal vestibule occurred as a long-term AE in 15% of the patients. However, long-term low-grade stenoses at the level of the nasal vestibule are frequent. They do also affect the patient and complicate the oncological clinical follow-up due to changes of the nasal passage and are therefore clinically highly relevant. Despite the high dose applied, no patient developed cartilage necrosis during follow-up, and the cosmetic results after combined RT with CIRT-B were excellent.

There is disagreement in the literature regarding prognostic factors for LC and survival after RT for malignancies of the nasal vestibule. Vanneste et al. found that increasing T classification was linked with poorer LC and LRC, and the risk of a local recurrence increased with tumor size (17). In a retrospective series of cancer of the nasal vestibule, Agger et al. found no statistically significant effects in 5-year LRC with regard to sex, age, or smoking status. However, Wang classification was prognostic for LRC and DSS in this series of patients (8). Kummer et al. could show that the effect of RT (DSS) is significantly correlated with tumor stage, and hence RT is less successful in T3 lesions. Limited success for T3 lesions should be interpreted with caution because only three patients were included, and no chemotherapy was applied in advanced stages (34). In a series published by Wallace et al., cause-specific survival was lower in patients with unfavorable T4 tumors (>4 cm with bone invasion) after definitive RT (35). A total of 56 patients with SCC of the nasal vestibule were treated and retrospectively evaluated by Langendijk et al. No significant association between Wang stage, tumor diameter, or tumor localization and LC was found by the authors (16). Our analysis did not identify prognostic factors related to LC and survival. This may possibly be explained by the small number of patients and the short follow-up.

Finally, the limitations of our analysis were the retrospective character and the limited patient number. Furthermore, patients had different tumor types, and 9.5% had resection alone during their treatment at initial diagnosis and RT was performed as salvage treatment. However, this is the first analysis reporting on CIRT-B combined with VMAT as an organ-preserving, primary procedure in malignant tumors of the vestibule or the anterior nasal cavity with involvement of the nasal vestibule and has a reasonable number of patients treated with a homogenous treatment approach.

## Conclusions

CIRT-B combined with VMAT with photons in the primary treatment of malignant tumors of the nasal vestibule and the anterior nasal cavity is safe and feasible, resulting in high local control and survival rates and thus is a good option as an organ-preserving therapy. In local or locoregional recurrences after definitive RT, there are good surgical salvage options for the patients.

No radiation-associated grade 4 or higher AE were documented, and the treatment was tolerated well. However, a relevant number of patients developed grade 3 acute AE mostly regarding the skin, mucosa, and swallowing at the end of treatment. A more limited proportion of patients developed late AE mostly at the paranasal sinuses or cisplatin-related hearing impairment that required medical interventions. Further investigations including the issue of potential target volume reduction within prospective trials on carbon ion beam irradiation in malignancy of the nasal vestibule and the anterior nasal cavity are warranted.

## Data Availability Statement

The raw data supporting the conclusions of this article will be made available by the authors, without undue reservation.

## Ethics Statement

The studies involving human participants were reviewed and approved by Ethikkommission des Fachbereichs Humanmedizin der Philipps-Universität Marburg. The patients/participants provided their written informed consent to participate in this study.

## Author Contributions

FE, SL, HH, and RE-C developed and planned the study. FE and SL performed data management. FE and SL performed data analysis. FE, SL, and RE-C performed data interpretation. FE and SL drafted the manuscript. RE-C, CD, US, FS, K-SB, BS, CL, AJ, and HH critically revised the manuscript for important intellectual content. All authors read and approved the final manuscript and agreed to be accountable for all aspects of the work.

## Conflict of Interest

The authors declare that the research was conducted in the absence of any commercial or financial relationships that could be construed as a potential conflict of interest.

## Publisher’s Note

All claims expressed in this article are solely those of the authors and do not necessarily represent those of their affiliated organizations, or those of the publisher, the editors and the reviewers. Any product that may be evaluated in this article, or claim that may be made by its manufacturer, is not guaranteed or endorsed by the publisher.

## Abbreviations

AE, Adverse events; CIRT, carbon ion radiotherapy; CIRT-B, carbon ion radiotherapy boost; CT, computed tomography; CTC, common toxicity criteria; CTV, clinical target volume; CTV-B, clinical target volume—boost; CTV-SIB, clinical target volume—simultaneously integrated boost; DSS, disease-specific survival; EBRT, external beam radiotherapy; ENI, elective nodal irradiation; EQD2, equivalent dose in 2 Gy; Gy, Gray; Gy(RBE), Gray (Relative biological effectiveness); GTV, gross tumor volume; GTV-B, gross tumor volume—boost; GTV-SIB, gross tumor volume—simultaneously integrated boost; IMRT, intensity modulated radiotherapy; IR, interventional/interstitial radiotherapy; LC, local control; LEM, local effect model; LRC, locoregional control; MRI, magnetic resonance imaging; OPS, organ-preserving survival; OS, overall survival; PFS, progression-free survival; PTV, planning target volume; RBE, relative biological effectiveness; RT, radiotherapy; SCC, squamous cell carcinoma; SD, single dose; TD, total dose; VMAT, volumetric intensity modulated arc therapy.
